# Disruption of retinol-mediated IL-6 expression in colon cancer-associated fibroblasts: new perspectives on the role of vitamin A metabolism

**DOI:** 10.18632/oncotarget.28399

**Published:** 2023-04-26

**Authors:** Romain Villéger, Marina Chulkina, Randy C. Mifflin, Don W. Powell, Irina V. Pinchuk

**Affiliations:** ^1^Université de Poitiers, UMR CNRS 7267, Ecologie et Biologie des Interactions, France; ^2^Department of Medicine at PennState Health Milton S. Hershey Medical Center, Hershey, PA 17033, USA; ^3^Department of Internal Medicine, Division of Gastroenterology and Hepatology, UTMB, Galveston, TX 77555, USA; ^4^Institute for Translational Sciences, UTMB, Galveston, TX 77555, USA; ^5^Department of Neuroscience and Cell Biology, UTMB, Galveston, TX 77555, USA

**Keywords:** tumor microenvironment, colon cancer, inflammation, fibroblasts, IL-6

## Abstract

Stromal myo-/fibroblasts (MFs) account for up to 30% of lamina propria cells in the normal human colon and their number is dramatically increased in colon cancer (CRC). Fibroblasts from cancers, also known as cancer-associated fibroblasts (CAFs), differ from normal colonic MF (N-MFs) and support tumor-promoting inflammation, in part due to increased IL-6 secretion. In this editorial, we highlight recent data obtained regarding IL-6 regulation in colorectal cancer CAFs through vitamin A (retinol) metabolism, discuss current limitations in our understanding of the mechanisms leading to the CAF pro-inflammatory phenotype, and discuss potential approaches to target CAF retinoid metabolism during CRC treatment.

## INTRODUCTION

Colon cancer is one of the most common malignancies and is a leading cause of cancer-related deaths worldwide [1]. While the tumor microenvironment (TME) supports tumor growth and immune escape through tumor-promoting inflammation [2], the mechanisms by which TME promotes CRC are far from being elucidated. Cancer-associated fibroblasts (CAFs) are very abundant in the TME and are among the major cells involved in tumor inflammation and progression.

We and others have previously demonstrated that CAFs are major producers of tumor-promoting cytokine IL-6 at T2-3 stage tumor in colon cancer [3–5]. IL-6 has been demonstrated to promote tumor cell proliferation, cancer stem cells, and metastasis in colon cancer [3, 4, 6]. While targeting IL-6 in the treatment of GI cancers is a promising strategy, the mechanisms involved in the increased expression of this cytokine are unknown. In a recent study [7], we reported that a profound decrease of alcohol dehydrogenase 1B (ADH1B) occurs in colonic stromal cells (known as fibroblasts or myofibroblasts) during the adenoma-carcinoma sequence and that ADH1B downregulation in CAFs contributes to the disruption of the retinol-mediated suppression of tumor-promoting IL-6 in neoplastic tissue. In this editorial, we discuss our findings and those from recent studies, with the aim of highlighting possible new translational research targets in CAFs involving vitamin A metabolism for CRC therapies deserving further investigation. We also discuss unpublished data that could provide perspectives in deciphering new mechanisms involved in vitamin A metabolic pathway and IL-6 production in CAFs.

### Decrease of retinol metabolizing enzyme ADH1B in colon cancer-associated fibroblasts promotes IL-6 production

In contrast to fibroblasts from normal tissues (N-MFs), CAFs promote tumor development and progression by providing cancer cells with cytokines and growth factors that support tumor growth [8]. Targeting CAFs has been recently suggested as a potentially novel therapeutic approach. In order to identify new CAF specific targets, the mechanisms behind the induction of a tumor-promoting activity in these cells must be understood. We pursued this strategy in our study [7] by isolating CAFs and N-MFs from patient tissues and assess their transcriptome. Cultured CAFs demonstrated significant changes in the network of genes involved in the immune responses, angiogenesis, regulation of cytokine signaling network and metabolism when compared to cultured N-MFs. Among the genes dysregulated in CAFs, change in IL-6 gene network was prominent leading to the increase in IL-6. Our data also suggested that alcohol dehydrogenase 1B (ADH1B), a class I alcohol dehydrogenase, was involved in the regulation of basal and inducible IL-6 in N-MF. This regulation was lost in colon cancer due to a dramatic decrease in the expression of ADH1B [7]. While the origin of the ADH1B abrogation in CAFs is still under investigation, Nadauld et al. reported that mutations of the human adenomatous polyposis coli (APC) gene could repress retinol dehydrogenases genes [9].

**Figure 1:**
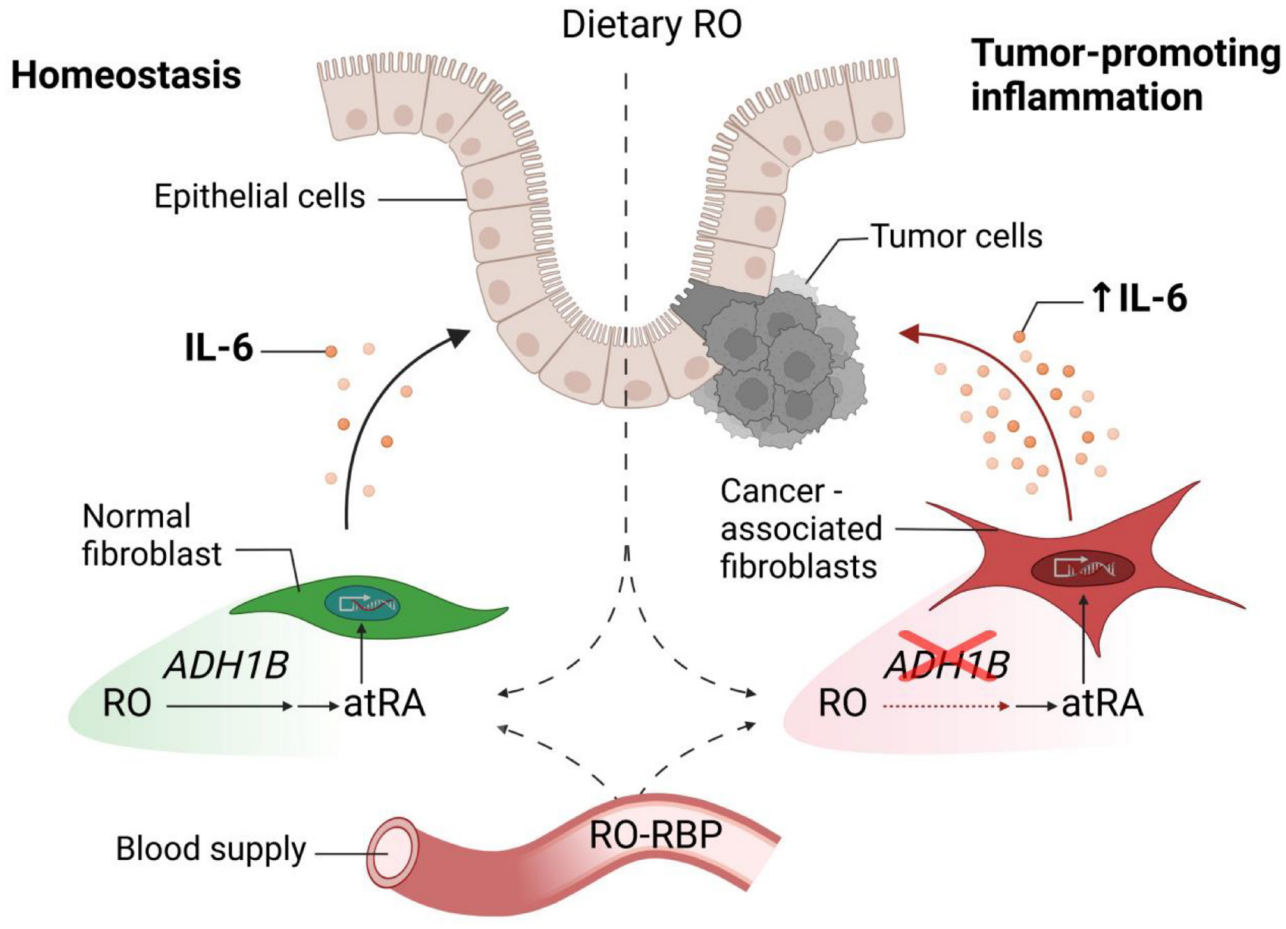
Retinol-mediated suppression of tumor-promoting IL-6 is disrupted in colon cancer-associated fibroblasts. CAFs have been reported as major cellular producers of tumor-promoting IL-6 in colorectal cancer. ADH1B, a key enzyme involved in the metabolism of retinoids and expressed in normal colonic fibroblasts, is expressed at low levels in CAFs. The critical suppression of ADH1B expression in LPS-stimulated CAFs could inhibit the conversion of RO into active atRA, which in turn contributes to IL-6 production in neoplastic tissues. Abbreviations: CAFs: cancer-associated fibroblasts; IL-6: interleukin 6; ADH1B: alcohol dehydrogenase1B; RO: retinol; atRA: all-trans retinoic acid; RBP: retinol binding protein.

Retinoids are known to inhibit tumor-promoting IL-6 production [10, 11]. ADH1B is among the enzymes involved in the metabolism of retinoids, converting retinol (RO) to retinaldehyde (RA), which is then further metabolized to all-trans retinoic acid (atRA). Further, a therapeutic anti-tumor effect of atRA has been proposed as a novel therapeutic approach for several cancers including colon cancer [12], but studies yielded controversial results [13]. We hypothesized that ADH1B may convert retinol to retinaldehydes which in turn could negatively regulate IL-6 production. Therefore, we next evaluated the consequences of the low levels of ADH1B in CAFs on IL-6 production after retinoid treatment and compared this to IL-6 production in N-MFs that exhibit high levels of ADH1B. In N-MFs, lipopolysaccharide (LPS) induced IL-6 production was inhibited by either RO or its byproduct atRA. However, although atRA effectively inhibited IL-6 production in CAFs, RO administration did not, confirming the inability of CAFs to utilize RO to suppress IL-6 production [7]. These results are summarized in Figure 1.

While the overall mechanisms responsible for the IL-6 increase within the CRC tumor stroma remain to be elucidated, our study highlights the crucial role of stromal vitamin A pathway in IL-6 regulation. However, a deficiency of atRA in CAFs may be not only due to *Adh1b* downregulation, but to alterations in several retinol metabolic enzymes, including retinol converting enzymes, retinaldehyde converting enzymes and atRA catabolic enzymes. These processes could result in lower retinol uptake/intracellular levels, with less conversion of retinol to atRA, and an increased catabolic rate of atRA [14–16]. Finally, the role of cancer associated microbiota should be considered as well in the disruption of retinol metabolism and the increase in tumor-promoting IL-6. Members of the alcohol dehydrogenase family metabolize a wide variety of substrates such as ethanol, retinol, and other aliphatic alcohols including, perhaps, those produced by the colonic microbiota. Additionally, Bhattacharya et al. has shown that microbiota-induced intestinal inflammation alters atRA metabolism, leading to a colonic atRA deficit and exacerbation of colonic carcinogenesis [17].

### A possible role of CYP26B1 in IL-6 regulation by retinoids

**Figure 2:**
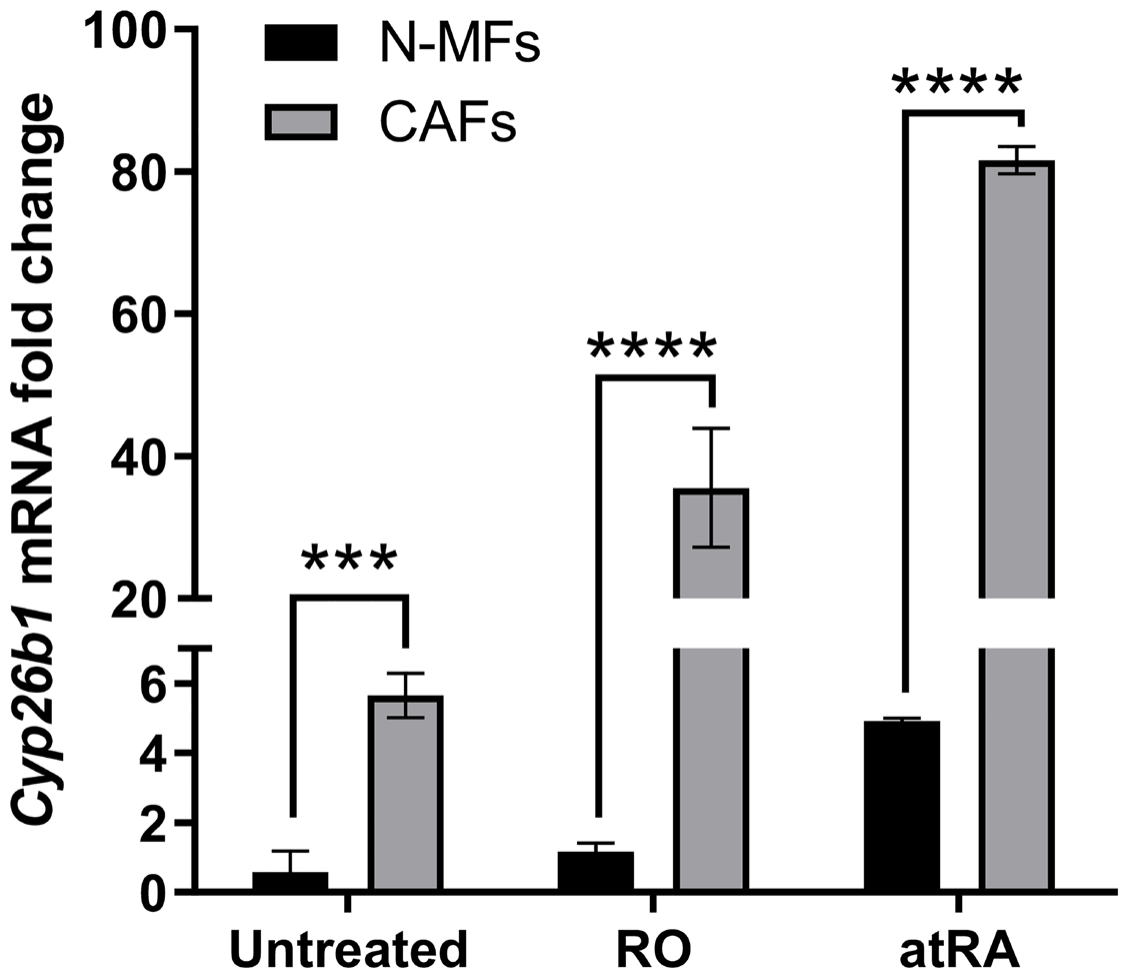
Cyp26b1 mRNA expression is higher in CAFs and is upregulated by RO and atRA. Cultured colonic fibroblasts have been isolated from normal mucosa or matched adenocarcinoma from two human biopsies and treated for 18 h with RO or atRA (2.5 μM). Effect of RO or atRA addition on Cyp26b1 mRNA expression was measured by RT-qPCR and normalized to β-actin in N-MFs and matched CAFs (two matched pair of fibroblast isolates were analyzed, 3 experimental repeats).

**Figure 3:**
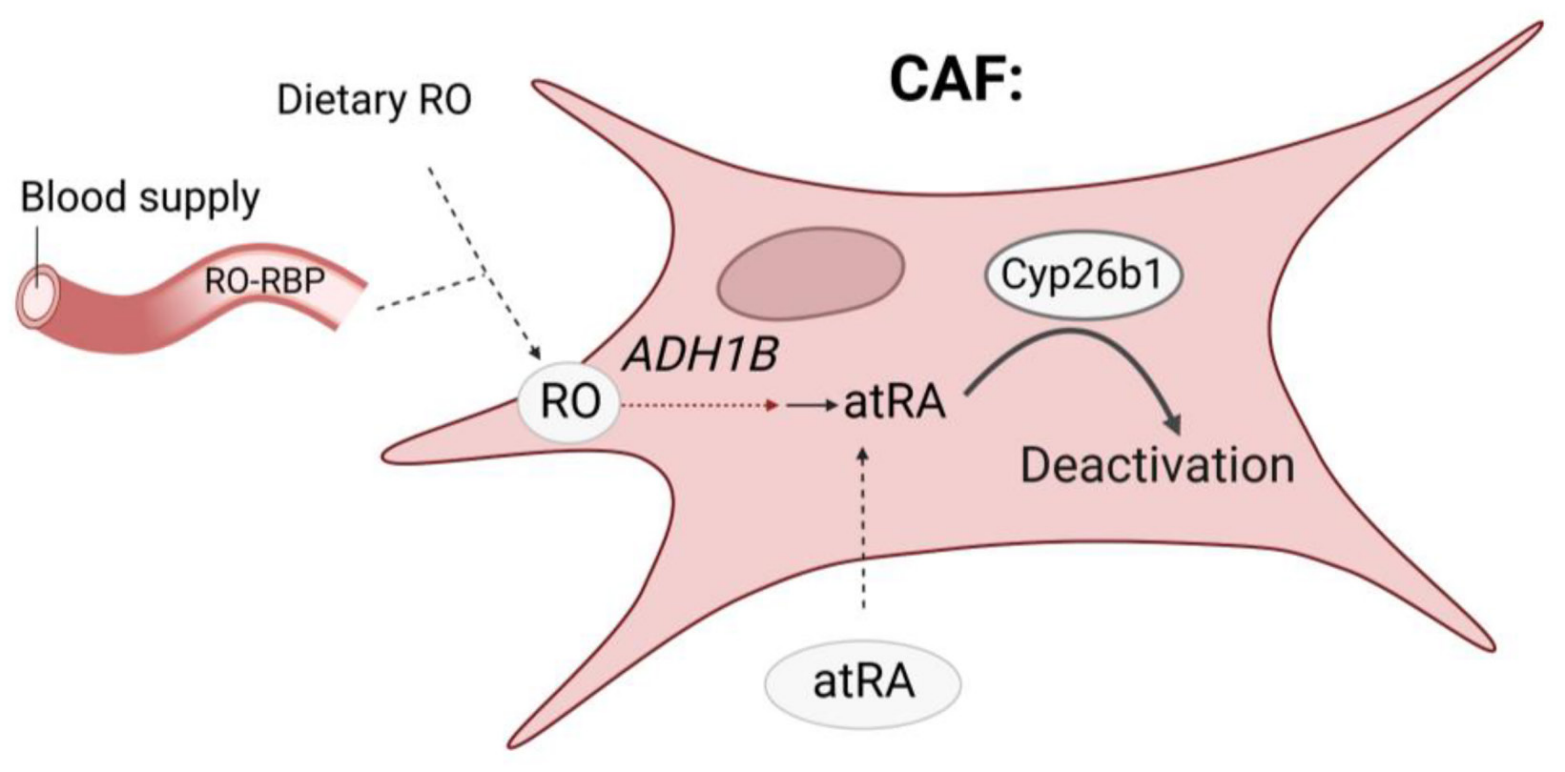
Preliminary data suggest that *Cyp26b1* over-expression in CAFs could decrease colonic atRA levels. Deactivation of atRA could participate to reduce its regulatory activity on IL-6 production.

Only a partial inhibition of IL-6 production in CAFs by exogenous atRA was observed in our experiments [7], suggesting that other pathways of IL-6 production exist in CAFs. One possibility is that levels of atRA may be altered by enzymes that affect atRA activity itself. Certain member of the P450 cytochrome family (CYP26A1, B1, and C1 enzymes) known to be induced by atRA itself are involved in deactivation of atRA [18, 19]. Brown et al. [15] have shown that these atRA metabolizing enzymes CYP26A1 and CYP26B1 are significantly overexpressed in colon cancer. Further, CYP26B1 is associated with poor prognosis in colon cancer [15]. We analyzed gene expression of *Cyp26a1*, *Cyp26b1* and *Cyp26c1* in N-MFs and CAFs isolated colon cancer patients. *Cyp26b1* was the predominantly expressed CYP26 enzyme in human N-MFs, while *Cyp26a1* and *Cyp26c1* were poorly detected (data not shown). Our unpublished data are in agreement with those from previous study showing that CAFs express higher basal level of *Cyp26b1* when compared to N-MFs isolated from a cancer-free area of colon (Figure 2) High *Cyp26b1* expression likely limits the inhibitory effect of atRA on IL-6 expression by reducing atRa levels in stromal cells. Indeed, our unpublished results demonstrate that 18 h of treatment with retinol or atRA results in a positive feed-back expression of *Cyp26b1* in MFs. The retinoid-induced level of *Cyp26b1* was higher in CAFs when compared to N-MFs (Figure 2), emphasizing the negative feed-back loop in which atRA induces its own metabolism in CAFs. This suggest that *Adh1b*^low^/*Cyp26b1*^high^ phenotype in CAFs could contribute to their resistance to retinoid treatment in order to decrease IL-6 expression (Figure 3). It has been previously reported that therapeutic use of atRA isomers is limited due to resistance to treatment emerging mainly from autoinduction of atRA metabolism [20]. Other investigators have used the combination of atRA and inhibitors of CYP26 enzymes (particularly CEP26A1) in order to improve efficacy of atRA treatment in some cancers. [21]. While an anti-inflammatory effect of CYP26 inhibitors has been reported for the topical treatment of skin inflammations [22], to date, the specificity of the inhibitors and their beneficial effects in cancer-associated inflammation have not been fully characterized. Thus, further investigation is warranted of a combination of atRA and a CYP26 inhibitor as a potential therapeutic approach for colon cancer.

Taken together, our data identified ADH1B as a novel mesenchymal suppressor of tumor-promoting IL-6 overexpression. Decrease/loss of ADH1B in MFs during the adenoma-carcinoma sequence contributes to disruption of the retinol-mediated suppression of tumor-promoting inflammation in CRC and to the increase of IL-6 in neoplastic tissue. To date, deeper investigations of IL-6 regulation by retinol metabolic pathways in CAFs are required to understand whether these pathways represent new CAF-directed targets for CRC treatment.
